# Development of an inducible lytic system for functional metagenomic screening

**DOI:** 10.1038/s41598-019-40470-4

**Published:** 2019-03-07

**Authors:** Jara Cárcel-Márquez, Amando Flores, Guadalupe Martín-Cabello, Eduardo Santero, Eva M. Camacho

**Affiliations:** Centro Andaluz de Biología del Desarrollo/CSIC/Universidad Pablo de Olavide/Junta de Andalucía. Departamento de Biología Molecular e Ingeniería Bioquímica, Seville, Spain

## Abstract

Functional metagenomic is a powerful tool that allows the discovery of new enzymes with biotechnological potential. During functional screenings of enzymes, the ability of the substrate to enter the surrogate host or the ability of this bacterium to export heterologous extracellular enzymes may hamper the technique. Here we have used an inducible autolysis system that lyses bacteria thus releasing its content in both, liquid and solid cultures, in response to anhydrotetracycline. The lytic cluster is tightly regulated to prevent impaired bacterial growth in absence of the inducer and produced very efficient though not complete bacterial lysis upon induction, which allowed the recovery of live bacteria. The system can be used in combination with specialised fosmids and *E. coli* strains that maximize transcription of metagenomic DNA. Our results show that colony-lysis on plates allows detection of an endogenous intracellular amylase activity naturally present in *E. coli* and clearly increased detection of clones coding for cellulase activities in a metagenomic screening, while allowing recovery of survivor positive clones from the lysed colonies in all cases. Therefore, this tool represents an important step towards the effective access to the extraordinary potential of the uncultivated bacteria genetic resources.

## Introduction

Culture-independent molecular analyses have revealed that most of the existing microorganisms in nature are still unknown to us^1^. This is because the great majority of them cannot be cultivated and characterised in a laboratory. Therefore, this extraordinary source of genetic information remains hidden even after extensive screening based on standard cultivation methods. To explore these sources, the metagenomic technologies^2^ involving direct isolation and cloning of DNA from environmental samples into suitable vectors have been developed. This offers ways of gene identification independent of the microorganism cultivation, either by sequence-based or function-based analysis, thus allowing more extensive analyses of the real diversity in nature.

While sequence analysis based on comparison with previously known sequences, has the inherent limitation that the genes identified are unlikely to be strongly divergent from the genes already known, functional metagenomic analysis, which is based on the detection of phenotypic changes in a bacterial host due to the acquisition of a metagenomic library clone, has the advantage of allowing the identification of new genes. However, functional metagenomic has two major limitations^3^.

On the one hand, the possibility of expression of metagenomic DNA in the surrogate host that maintains the DNA library is low. To circumvent this limitation a number of broad host-range vectors for functional screening in a number of different gram negative bacteria have been developed, along with expression systems to allow transcription of long stretches of environmental DNA from vector promoters^3^. Of particular interest are those that incorporate viral components of the transcriptional machinery, which support transcription even across transcriptional terminators potentially present in the environmental DNA^4^.

On the other hand, the identification of clones with the activity of interest requires the development of highly sensitive and cost-effective screening strategies to detect clones displaying even low levels of activity among the many thousands screened. Direct detection on agar plates has the advantage that it can be carried out without the need for any special equipment. Usually, the identification of positive clones consist of a phenotypic screening based on changes in the morphology or colour of the colony, or the presence of clear halos around colonies grown on particular differential media^3^. Therefore, the selection of clones depends on the detection of visible phenotypes, not always easy to see, among the myriad of negative colonies. Detection is also limited by the ability of the substrate of intracellular activities to enter the bacterial cells, or the ability of the surrogate host to export extracellular enzymes.

For example, most of the polymer-hydrolysing enzymes are extracellular enzymes and, although *E. coli* has been the most frequently used host for functional screening, efficient secretion of metagenomic enzymes of this type by *E. coli* is unlikely. Thus, so far, the identification of these hydrolysing activities directly from *E. coli* colonies probably relies on the spontaneous lysis of a small percentage of cells within the colony to release their contents to the medium. Accordingly, any procedure increasing cell permeability without compromising enzyme activity and compatible with the recovery of live bacteria from the positive colony is expected to increase the frequency of positive hits for such non-secreted extracellular enzymes.

In this work we have used a metagenomic library constructed using fosmid pMPO579 and the specialized strain MPO554^4^. The plasmid pMPO579 is a fosmid vector that contains an *oriT* to be transferred by conjugation, along with a phage T7 RNA-polymerase-dependent promoter and a salicylate-inducible p*sal* promoter followed by a *nut*_*L*_ site, whose transcription is subjected to transcription antitermination by the lambda phage antitermination protein N. Besides its own *ori*2 replication origin of F plasmids, the fosmid also has a second broad-host range RK2 replication origin engineered for arabinose-inducible copy number. The specialised *E. coli* strain also produces the N antitermination protein in response to salicylate, thus allowing processive heterologous gene transcription across the metagenomic DNA fragment. This system has already been successful in identifying antibiotic resistance determinants^4^ and other functions of interest^5^ that would not have been detected with other methods available.

To circumvent the aforementioned limitation in the detection of activities that require substrate transport or protein secretion we have used a previously developed inducible autolysis system based in the lysis operon of the lambda phage. This device provokes lysis of *Salmonella* and *E. coli* cells in response to the addition of anhydrotetracycline (AHT). Despite massive lysis causing the release of the bacterial cell contents to the growth medium, a small fraction of surviving bacterial cells consistently remains after the induction of lysis^6^. Here we have adapted this system for its use in functional metagenomic analyses and have evaluated its usefulness as a tool for increasing detection of positive clones in agar plate assays. The results corroborate that the inducible autolysis system does not affect bacterial viability in the absence of induction, but efficiently lyses cells upon induction. Release of cell contents by this method permits the detection of intracellular enzymatic activities not identified otherwise and improves detection of extracellular enzymatic activities while still allowing the recovery of live positive clones.

## Results

### Construction of a compatible AHT-inducible lysis system and characterisation of bacterial lysis in cultures

Although an AHT-inducible lysis system was developed in a previous work^6^, the set of plasmids available is not compatible for their use in combination with the fosmid of the metagenomic library due to antibiotic resistance marker incompatibility. In order to make both (fosmid and autolytic plasmids) compatible, we transferred the lysis system to a multi-copy vector that confers resistance to ampicillin. To that end the *SRRz* cluster of lambda phage was cloned under the P_*tet*_ promoter control in pMPO1070 (a derivative of pASK-IBA43) generating plasmid pMPO1077. The lysis gene cluster consists of four genes: *S* encoding the holin that produces a collapse of the membrane potential and permeabilisation of the cell membrane^7^; *R* encoding the endolysin degrading the peptidoglycan layer; and *Rz* and *Rz1* whose products form a complex that bridges the cell and outer membranes, causing the disruption of the latter^8^.

Both set of plasmids, the previously developed low copy vectors pMPO1632 and its control pMPO1631 (lacking the lysis system), and the new high copy version pMPO1077 and its control pMPO1070 were transferred to *E. coli* DH5α and bacterial culture lysis was characterized.

**Figure 1:**
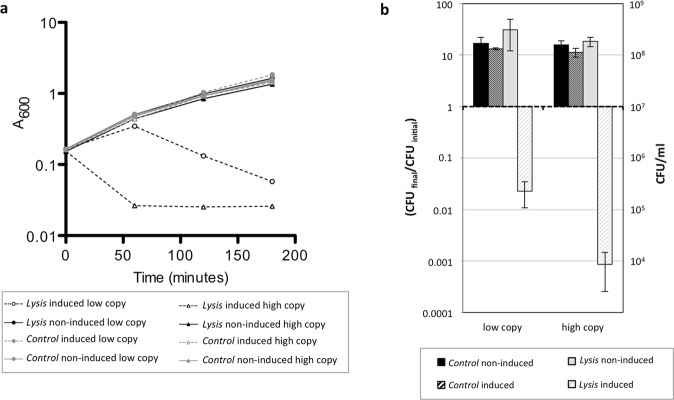
Bacterial lysis phenotype in liquid cultures. (**a**) Growth of bacterial cultures of DH5α carrying lysis (pMPO1632 high copy or pMPO1077 low copy) or control plasmids (pMPO1631 high copy or pMPO1070 low copy). When the optical density of the cultures reached 0.2 they were divided in two groups and AHT was added in half of the cultures (time 0). (**b**) Change in bacterial population after induction. The number of viable cells was determined by counting colony forming units at time 0 and at the end of the experiment. Graphics represents the mean ± SD of three independent cultures.

The results indicated that in absence of AHT, cultures continued proliferating independently of presence of the lysis system (Fig. 1a). However, the A_600_ of bacterial cultures carrying the lysis systems decreased after AHT induction in both, high and low-copy vectors. Interestingly, in the high copy vector the lysis began earlier (apparently at the time of induction) and was faster than the lysis observed from low-copy vector. The number of colony forming units (cfu) on LB plates decreased 100-fold when compared to the cfu at the time of induction for the low-copy vector and more than 1000-fold for the high-copy vector. However, in both cases a fraction of the bacterial population survived (Fig. 1b). The bacterial survival rate was 1 and 0,1% of the population at the time of induction in low- or high-copy vector cultures, corresponding to approximately 10^5^ cfu mL^−1^ and 10^4^ cfu mL^−1^, respectively. The results indicated that the lysis obtained from high copy vectors is much faster and efficient but still allows recovery of viable bacteria after lysis induction.

### Bacterial lysis and recovery of viable bacteria in solid media

**Figure 2:**
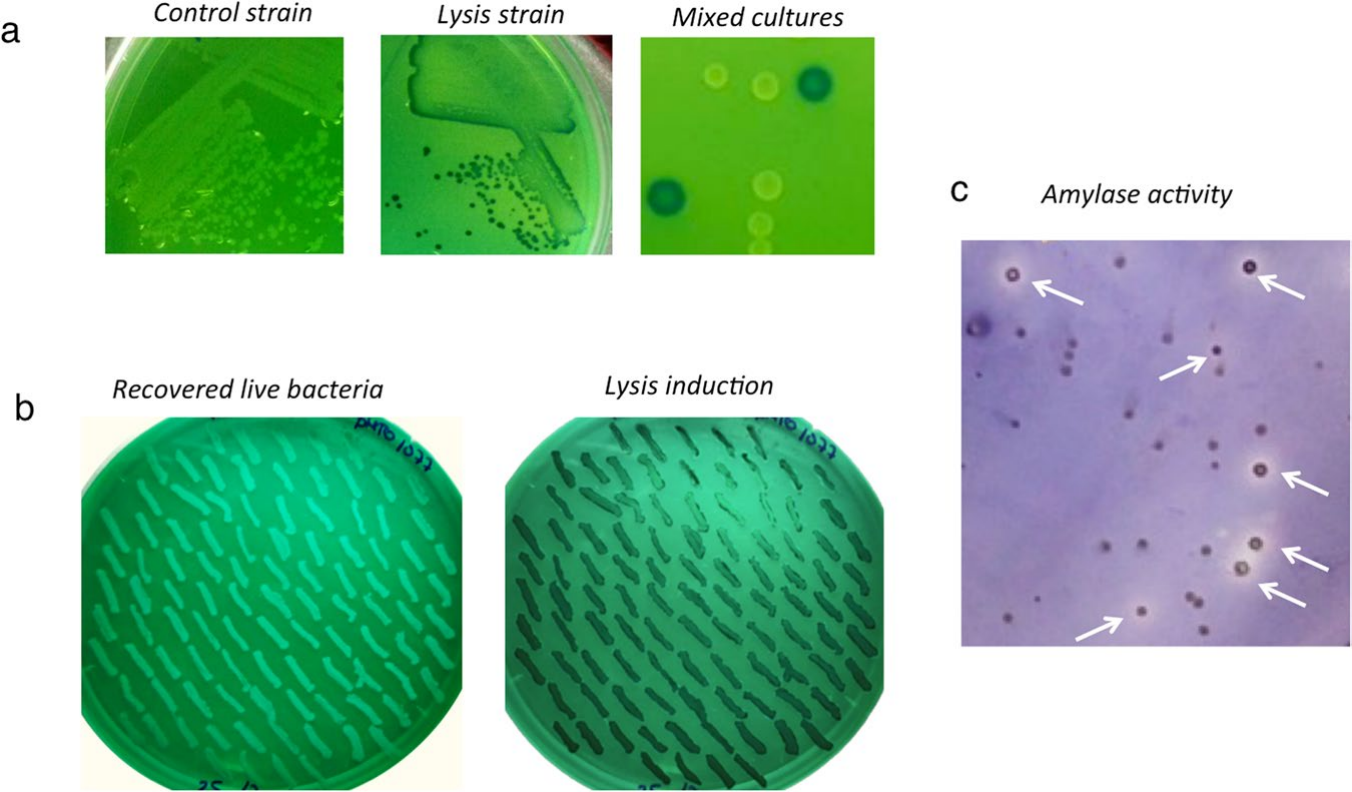
Bacterial lysis phenotype in colonies. (**a**) Bacterial lysis in EBU plates. Lysis induction of MPO554 Nal^R^ colonies bearing plasmids pMPO1070 (left panel), pMPO1077 (central panel) or mixed cultures (right panel) grown on EBU plates. (**b**) Recovery of live bacteria after lysis. On the left, recovery of live bacteria from lysed colonies picked through the soft agar layer from EBU plates and grown again on EBU plates. On the right, subsequent lysis induction of survivors recovered previously. (**c**) Detection of the endogenous amylase activity of *E. coli*. Mixed cultures of MPO554 carrying the plasmid pMPO1077 or pMPO1070 were grown on LB plates supplemented with starch. When the colonies were visible, lysis was induced and the plates subsequently stained to reveal the amylase activity. The white arrows indicate the colonies that carry the lysis plasmid.

The data obtained in liquid cultures suggested that lysis and recovery of live bacteria in solid media should be possible. To test this hypothesis, we induced lysis of colonies grown on EBU plates, which are normally used for detection of pseudolysogens in P22 transduction protocols^9^. This medium contains dyes that stain colonies containing lysed bacteria. In EBU plates, colonies containing lysed bacteria produce dark blue colonies while regular colonies are white/light-green. After growth of colonies on EBU plates, lysis was induced by overlaying the EBU plates with soft agar containing AHT. Incubation at 37 °C was resumed and the plates were monitored for colony lysis. As shown in Fig. 2(a), after AHT induction, bacteria carrying the control (empty) vector remained white/light-green while colonies formed by bacteria bearing the lysis plasmid became dark blue 4 h after AHT addition, indicating bacterial lysis. It is important to note that in the presence of lysis, the appearance of white/light-green colonies was never observed, indicating that the lysis system worked very eficiently in all the colonies, and mutant colonies immune to lysis were not detected in the experiments carried out in this work. Subsequently, live bacteria from lysed colonies were recovered by picking colonies through the soft agar layer and streaking out onto EBU plates. We obtained live bacteria from 100% of the picked colonies (Fig. 2b) and all of them maintained their ability of autolysis in response to inducer as shown by subsequent lysis of the recovered bacteria (Fig. 2c).

### Bacterial lysis and detection of an intracellular activity

To determine if lysis on solid media is sufficient to allow detection of an intracellular activity, we tested if the lysis system promoted detection of the endogenous amylase activity naturally present in *E. coli*^10^ that is not excreted to the medium. Raha *et al*.^10^ demonstrated that *E. coli* produces a cytoplasmic alpha-amylase, AmyA, and showed that this enzyme remains inside the bacteria and does not digest extracellular starch unless the cells are lysed. To test the suitability of the lysis system to detect this activity, dilutions of mixed cultures of MPO554 (which expressed the endogenous amylase) containing 1:10 lysis *vs* control strains, were plated on LB plates containing starch. After colony growth, lysis was induced and plates were incubated overnight at room temperature. A lugol solution^11^ was subsequently poured over the plates to reveal amylase activity. Pale blue, purplish or colourless halos surrounding colonies (in contrast to the blue colour of negative controls) indicated the presence of the activity. As shown in Fig. 2C, after AHT induction, the lysis system allowed detection of the amylase activity in some colonies. To distinguish between colonies bearing the lysis and the control vectors, positive and negative colonies were picked through the soft agar layer and lysis was tested in EBU plates (see Supplementary Fig. S1). The results indicated that the presence of lysis allow detection of amylase activity while the activity was not revealed in control strains MPO554 bearing the empty vector.

### Using the lysis system for the screening of cellulase activity in a metagenomic library

**Figure 3:**
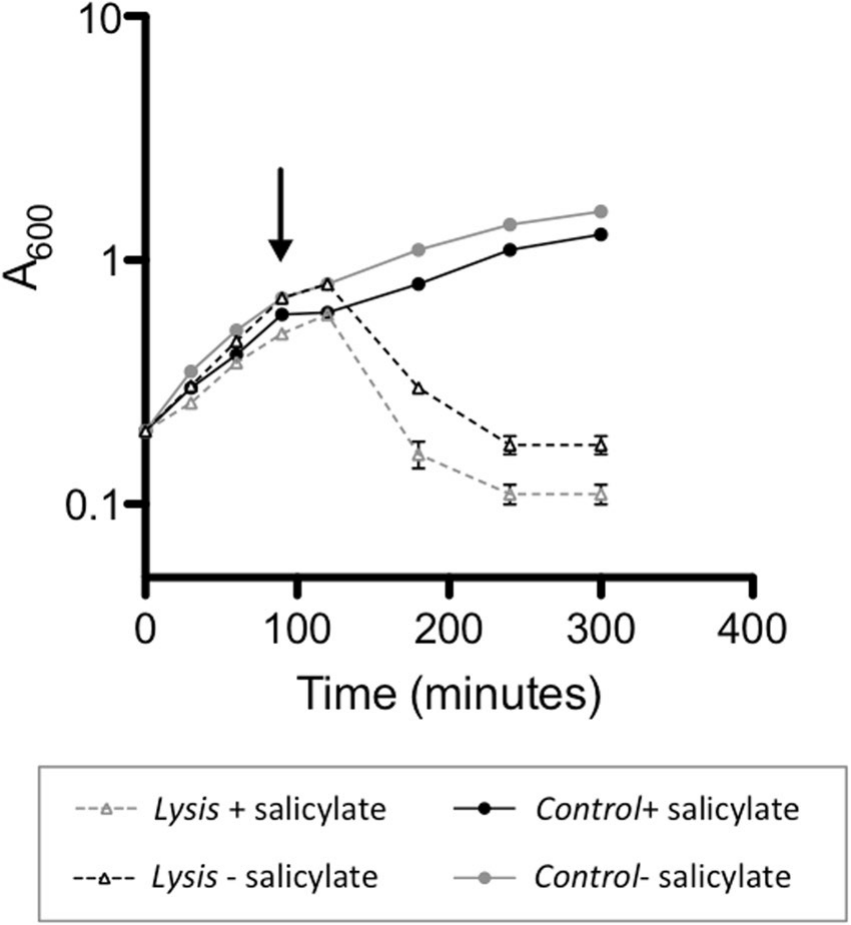
Combination of heterologous expression and lysis systems in bacterial cultures. Growth of bacterial cultures of MPO554 Nal^R^ harbouring lysis (pMPO1077) or control plasmids (pMPO1070) and the plasmid pMPO579 for the heterologous expression of metagenomic DNA in response to salicylate. Overnight cultures grown in LB, were diluted in fresh medium and once the A_600_ of the cultures reached 0.2, they were divided in two groups, salicylate was added in half of the cultures and after 90 minutes of growth AHT was added to both groups (salicylate induced and non-induced) (time indicated by the arrow). Graphics represents the mean ± SD of three independent cultures.

The expression of the metagenomic DNA in our specialised fosmid (pMPO579) and strain (MPO554) is induced by salicylate. For the use of the autolytic system in combination with this fosmid and strain it is necessary that the lysis can be induced while the heterologous DNA is being expressed. To test this, we measured lysis induction in cultures of MPO554 bearing plasmids pMPO579 and the lysis (pMPO1077) or control plasmid (pMPO1070), after 90 minutes of growth in the presence or absence of salicylate. As shown in Fig. 3, the previous induction of heterologous transcription with salicylate did not impair the lysis induction with AHT and, therefore, both systems are compatible and can be used sequentially.

To evaluate the system in a real screening, we decided to look for cellulase activity in a metagenomic library previously constructed in our laboratory. The metagenomic library consists of 6.5 Gb of metagenomic DNA of a petroleum-contaminated soil and was constructed in a fosmid vector that promotes heterologous expression of metagenomic DNA after salicylate induction in the specialized strain MPO554^5^. The constructed library consists of 185,000 independent clones maintained in the *E.coli* strain EPI300-T1.

For the screening, the metagenomic library was transferred by triparental mating^12^ to the MPO554 Nal^R^ strain^4^ carrying the lysis plasmid (pMPO1077). The mating mixture was diluted on LB selective medium containing 1% of carboxymethyl cellulose (CMC) to obtain 10^2^ to 10^3^ cfu of transconjugant cells per plate (15-cm diameter). The plates also contained salicylate to induce heterologous transcription and arabinose to increase the fosmid copy number. After colony growth, we induced lysis by overlaying the plates with soft agar containing AHT, as above. The plates were further incubated overnight at room temperature. Subsequently, cellulase activity was detected by Congo red staining^13^. Radial diffusion of enzyme produces zones of hydrolysis, which can be visualized by staining the non-degraded substrate with Congo red dye. Approximately 200.000 colonies distributed across 150 plates were screened.

After detection of putative positive clones, survivors from the colonies were picked out through the soft agar layer and cellulase activity was confirmed as before: firstly, by growing the positive colonies on LB plates supplemented with CMC, salicylate, arabinose and antibiotics; secondly, by inducing subsequent lysis with AHT and Congo red staining. Finally, we confirmed 13 positive clones out of the 50 initially detected.

**Figure 4:**
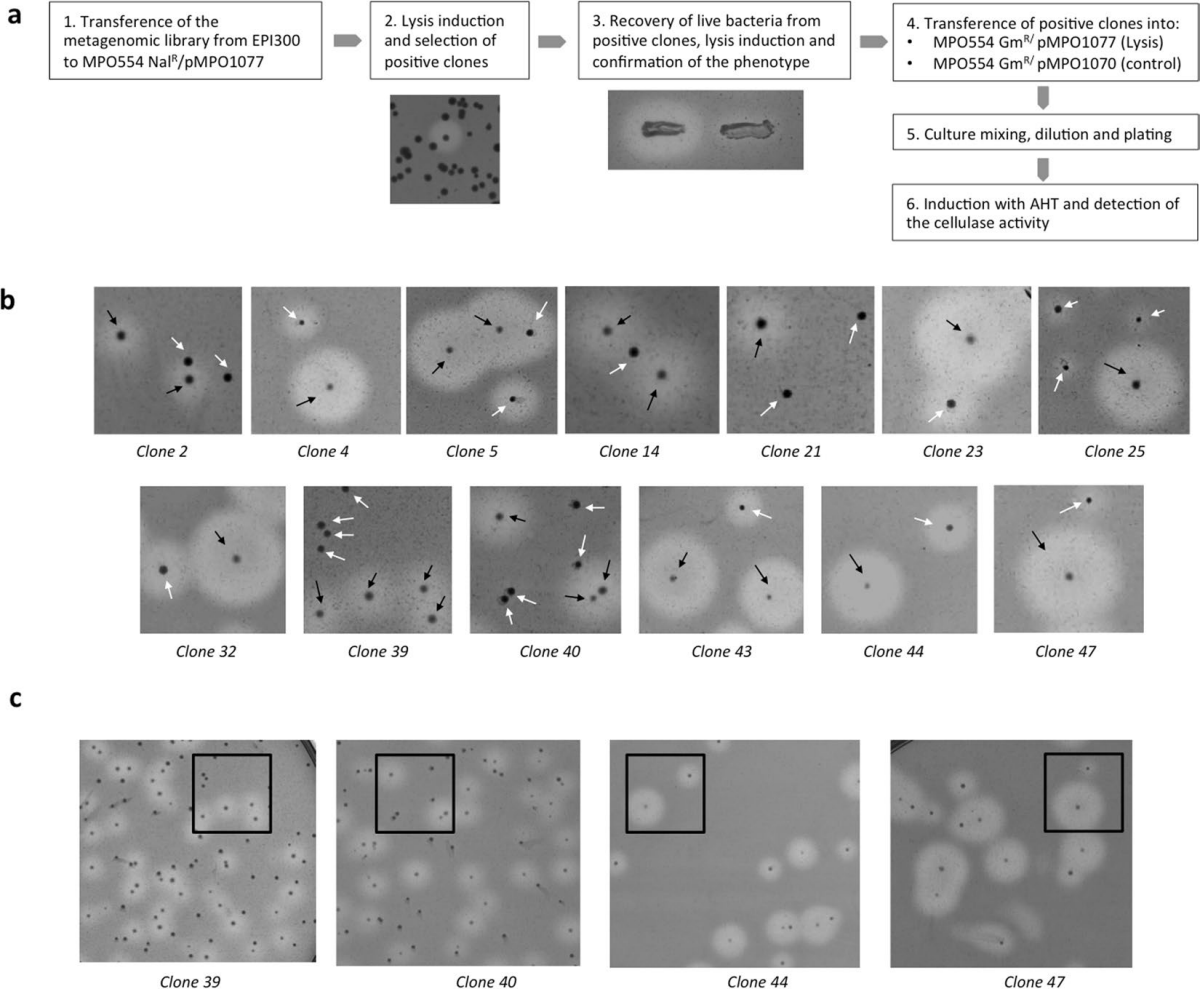
Screening of cellulase activity. (**a**) Schematic representation of the methodology used to screen for cellulases in metagenomic libraries in combination with the lysis plasmid. (**b**) Cellulase activity of positive clones in the presence of lysis (black arrows) or control plasmids (white arrows). (**c**) A zoomed-out picture of some clones; the black squares indicate the area shown in b.

To determine if the lysis system improves the cellulase activity detection, we tested the activity of the positive clones in the presence or in the absence of lysis. To this end, we transferred the positive clones by triparental mating from MPO554Nal^R^/pMPO1077 to MPO554 Gm^R^ /pMPO1077 and MPO554 Gm^R^ /pMPO1070 as control. Mixed cultures of both strains (MPO554 Gm^R^ with lysis or empty vector) were diluted and spread in ratio 1:1 onto LB plates containing CMC, arabinose, salicylate and the appropriate antibiotics. As before, after colony growth, lysis was induced and cellulase activity was revealed with Congo Red. As shown in Fig. 4, about half of the colonies of clones 2, 14, 21, 39 and 40 did not show any halo whilst for the remaining clones, colonies showed halos displaying two clearly different sizes. To distinguish between colonies bearing the lysis and the control vectors, colonies with different phenotype (large halo *versus* small or no halo) were picked through the soft agar layer and lysis was tested in EBU plates (see Supplementary Fig. S1). All colonies showing a large halo bore the lysis system whilst all the ones showing no halo or a small halo had the control plasmid without the lysis system. This indicates that in the absence of lysis, 5 positive clones would have been completely undetected and identification of many the remaining ones could have been hampered by the small size of the halos. Therefore, we conclude that induction of the lysis system significantly improved the detection of cellulose positive clones in all cases.

## Discussion

Since enzyme-based processes are less expensive, more efficient and cleaner than chemical processes, microbial enzymes are used in almost all industrial sectors^14^. The use of functional metagenomic techniques for the discovery of new classes of enzymes with biotechnological potential is a powerful tool that allows us to select potentially interesting enzymes based on their activity. Reports of functional screening for polymer hydrolysis activities have increased in recent years, such that these are emerging as some of the enzymes most frequently identified by this method^14–16^. Direct screening of colonies on agar plates based on phenotypic changes in colonies is the simplest way of analysing function. This strategy is particularly useful when searching for hydrolytic enzymes forming degradation halos on appropriate substrates. One of the limitations of this approach is the ability of the expression host of the metagenomic library to secrete the enzyme to the culture medium to access the substrate.

*E. coli* has limited protein secretion capacity, which may hamper the discovery of this type of enzymes. Despite this drawback, this bacterium has been the surrogate host most frequently used for functional detection due to its fast growth and the availability of tools for genetic screening and manipulation^3,14,15^. Probably, the identification of intracellular activities directly from colonies is possible because a small percentage of cells within a colony die and release their content. To improve the sensitivity of the assay, different treatments have been described that increase cell permeability that should increase the frequency of positive hits for non-secreted enzymes^17–19^. Such treatments include enzymatic lysis and/or freezing and thawing^17^, treatment with permeabilising chemical agents^17,18^ or UV-inducible autolytic vectors^19^. The autolytic system by Li *et al*.^19^, consist of the lambda lysis cassette under the control the UV-inducible *umuDC* promoter. This system has not been tested in colonies and has two main limitations: first, the use of UV as the inducer that can produce mutations and second, the efficiency of lysis is only approximately 60% at 30 °C and erratic at 37 °C.

Here, we have developed a new autolytic system also based in lambda lysis cluster but transcriptionally controlled by the tetracycline promoter/operator inducible by tetracycline or its non-toxic analogue anhydrotetracycline. The *tet* system shows a large regulatory window with high expression rates in the presence of AHT as the inducer and an extremely low basal expression level in the repressed state^20^. This makes this system suitable for controlling the expression of genes encoding poisonous products such as an autolytic cluster, as we have demonstrated previously^6^.

We have shown that this autolytic system induced by AHT works in *Salmonella*^6^ and different strains of *E. coli*, including the specialized strains designed in our laboratory, which, in combination with the appropriate vectors, maximize the transcription of heterologous genes of the metagenomic DNA^4^. The efficiency of lysis, measured as bacterial death, is undetectable under repression conditions but close to 100% in the presence of AHT, with a survival rate lower than 10^−3^. This means that almost 99,9% of bacterial population is lysed after AHT induction but also that, even in liquid cultures, a small amount of bacteria survive. Lysis can also be induced in colonies grown on solid media and we have demonstrated that bacterial death is accompanied by release of sufficient bacterial content to the agar plate to allow detection of an endogenous and natural (non-overexpressed) intracellular activity present in *E. coli*, such as the amylase activity.

However, in spite of the high efficiency of lysis, the survival percentage was sufficiently high to allow recovery of live bacteria from every single colony tested. Interestingly, survival was not due to loss of the plasmid or mutations in the lytic system since the survivors maintained their sensitivity to lysis in the presence of AHT. Whether this stochastic possibility of survival is due to the regulatory system by tetracycline or to the intrinsic dynamics of the lysis system it has not been explored, but for its application in functional metagenomic screening, this supposes an added advantage given that, after the initial screening, we can reuse the lytic system present in the positive hits to confirm the phenotype. The confirmation of putative positive clones is very important to rule out false positives that may occur during the screening.

We have tested the system in a real screening for cellulases using a metagenomic library constructed previously in our laboratory from a petroleum-contaminated soil^5^. We tested the system in combination with specialised fosmid and strains developed in our laboratory^4^. We transfer by conjugation the metagenomic library to the specialised strain in which we had previously introduced the autolytic system.

Our results suggest that the presence of the lysis system does not adversely affect the mating frequency compared to the previous data^4^ (data not shown) and allows the growth of colonies in the presence of arabinose to increase the fosmid copy number and salicylate to promote heterologous transcription. Even under these conditions of induction of metagenomic DNA expression, the addition of AHT induces the production of sufficient amount of lytic protein to lyse bacterial cells in liquid cultures and colonies on agar plates. The lysis of the colonies results in the release of the bacterial content to the agar medium and significantly improves the ability to detect positive clones.

Our autolytic system combined with fosmid and strains that maximize metagenomic DNA expression improves significantly the sensitivity of the screening procedures based on phenotypic changes of colonies on agar plates for the search of extracellular activities. This system is also compatible with many of the fosmids and vectors normally used for the construction of metagenomic libraries^3^ and could be combined with previously constructed metagenomic libraries simply by transferring the library to recipient strains carrying the lysis plasmid or alternatively by electroporation of the lysis plasmid to cells hosting the metagenomic library. This new system is therefore a powerful tool for functional metagenomic analyses, less expensive and more efficient than others described before.

## Methods

### Molecular biology general procedures

DNA manipulations were performed according to standard procedures^21^. The *SRRz* (1917 pb) cluster was obtained by PCR amplification of lambda DNA using DNA primers lisFw (5′-ACGGATGGCAACATATTAAC-3′) and lislabdrev (5′-ATATGGGCAACTCTATCTGC-3′) and cloned under the P_*tet*_ promoter of pMPO1070 derivative of pASKIBA43. The plasmid pMPO1070 derived from pASKIBA43 to which the strong RBS sequence and the 6xhistidine tag was removed by digestion with NdeI and XbaI and subsequent filling of 5′-protruding ends with Klenow and religation. Plasmid pMPO1070 containing the *SRRz* under the P_*tet*_ promoter control was named pMPO1077.

### Construction of gentamicin resistant derivative of the strain MPO554

To integrate the gentamicin resistance gene into the chromosome of MPO554 a modification of the Datsenko procedure^22^ was used. The gene was amplified by PCR from pBBR1MCS5^23^ using the previously phosphorylated primers semiFRTGmFw (5′-GAAAGTATAGGAACTTCTGGACGCACACCGTGG-3′) and semiFRTGmRev (5′- TAGAGAATAGGAACTTCCTGGCGGCGTTGTGAC-3′). The product of PCR was religated and subsequently electroporated into MPO554 bearing the plasmid pCP20^24^. pCP20 has temperature-sensitive replication and thermal induction of the FLP recombinase. MPO554 contains a FRT sequence into the chromosome recognized by FLP recombinase allowing site-specific recombination. MPO554/pCP20 was transformed with the religated product of PCR indicated above and Gm^R^ transformants were selected at 37 °C. After colonies growth we tested loss of pCP20 and selected Gm^R^, Ap^S^ and Cm^S^ colonies. Integration of the gentamicin resistance gene into the FRT sequence was confirmed by PCR amplification using primers SacP1 and trgEC-E2^4^.

### Bacterial strains and growth conditions

The bacterial strains and plasmids used in this study are described in in Supplementary Table S1. Luria-Bertani (LB) medium or LB-agar was used as the standard growth medium, and bacteria were grown aerobically at 37 °C except for the screening and selection of fosmids conferring cellullase activity that were carried out at 30 °C. When necessary, antibiotics were used at the following concentrations: 12.5 µg mL^−1^ chloramphenicol, 15 µg mL^−1^ nalidixic acid, 10 µg mL^−1^ gentamicin and 100 µg mL^−1^ ampicillin. The copy number of the fosmids in EPI300TM-T1 and derivative strains was increased by the addition of 1 mM arabinose. 1 mM salicylate was used as an inducer of heterologous expression for functional screenings in plates.

For amylase activity screenings, bacteria were grown in LB containing starch 0,5% and supplemented with appropriated antibiotics. To reveal the amylase activity after colonies growth and lysis (see below) plates were stained with lugol solution (Sigma) and the development of colour was observed. Pale blue, purplish or colourless halos indicated the positive activity while blue colour indicated negative colonies.

For cellulase activity screenings, bacteria were grown in LB containing 1% of carboxymethyl cellulose (CMC) and supplemented with appropriated antibiotics and arabinose and salicylate (see below). After colonies growth and lysis (see below) plates were stained with an aqueous solution of 10 mg mL^−1^ Congo red for 30 minutes^13^. The stain was then poured off and the plate washed with 1 M sodium chloride (30 minutes). A clear halo surrounding colonies indicates a cellulase producing clone.

### Lysis induction

For lysis induction we used the protocols previously described by Camacho *et al*.^6^. For lysis of liquid cultures, bacteria were grown at 37 °C in LB supplemented with the appropriate antibiotics and supplements. After growth, the saturated cultures were diluted in LB and allowed to grow until reaching an optical density (A_600_) of 0.2, at this point each culture was divided into two groups. In one of the duplicate groups, lysis was induced with the addition of AHT at a final concentration of 0.2 μg mL^−1^ while the other set of cultures was the non-induced control group. Both sets of cultures were incubated at 37 °C and the A_600_ were measured at regular time intervals. To quantify the number of viable bacteria, cultures dilutions were spread on LB plates without antibiotic and colony-forming units (CFU) were counted.

For induction of expression with salicylate and subsequent lysis induction cultures of MPO554 Nal^R^ cultures of MPO554 NalR carrying the appropriate combination of plasmids were grown at 37 °C in LB supplemented with antibiotics. Once grown, cultures were diluted in LB supplemented with arabinose 1 mM and, and once an optical density (A_600_) of 0.2 was reached, each culture was divided into two groups. One of the duplicated groups was induced with salicylate while the other set of cultures were the non-induced group. Both sets of cultures were incubated at 37 °C and the A_600_ was measured at regular time intervals. After 90 minutes of growth at 37 °C, autolysis of each culture was induced with AHT to a final concentration of 0.2 μg mL^−1^.

For lysis of colonies in solid media, once the bacteria are grown on the appropriate plates, autolysis was induced by pouring AHT to a final concentration of 0.2 µg mL^−1^ in a layer of soft agar (LB with agar 8 mg mL^−1^) and plates were incubated at 37 °C until lysis was observed or o/n at room temperature.

### Conjugative matings

Vectors and metagenomic libraries were transferred by triparental matings^12^ with DH5α/pRK2013 as the helper strain. Conjugative matings were performed on LB-agar without antibiotic selection overnight at 30 °C. The mating mixtures were then plated on LB-agar with the necessary antibiotics for the transconjugants selection, salicylate for inducing heterologous expression and arabinose for increasing the copy number of the fosmid, and incubated at 30 °C for 24 h.

Mating frequencies were estimated as the ratio of transconjugant clones of the recipient strain (chloramphenicol/nalidixic resistant or chloramphenicol/gentamicin acid resistant clones) to total clones of the recipient strain (either gentamicin or nalidixic acid resistant, depending on the recipient strain).

A more detailed protocol for the use of the lysis system in a metagenomic screening of cellulases is provided in Supplementary Methods.

## Supplementary information

Supplementary material

## Data Availability

All data generated or analysed in this study are included in this article.
