# Supplementary material for: Development of an inducible lytic system for functional metagenomic screening
Source: Sci Rep. 2019 Mar 7;9:3887. doi: 10.1038/s41598-019-40470-4 (PMC6405747; doi:10.1038/s41598-019-40470-4)
Supplement: Supplementary file 1 — Supplementary material [file 41598_2019_40470_MOESM1_ESM.pdf]

Development of an inducible lytic system for functional metagenomic screening

Jara Cárcel-Marquez, Amando Flores, Guadalupe Martín-Cabello, Eduardo Santero, Eva M. Camacho*

Centro Andaluz de Biología del Desarrollo/ CSIC/ Universidad Pablo de Olavide/ Junta de Andalucía. Departamento de Biología Molecular e Ingeniería Bioquímica, Seville, Spain

Supplementary Methods

Functional metagenomic screening of cellulases using the lysis system

The metagenomic library used consisted of 185,000 independent clones maintained in the *E.coli* strain EPI300-T1 [1]. For the screening, the metagenomic library was transferred by triparental matings [2] with DH5α/pRK2013 as the helper strain to the MPO554 Nal^R strain [3]) carrying the lysis plasmid (pMPO1077). Mating frequencies were previously estimated as the ratio of transconjugant cfu of the recipient strain (chloramphenicol, ampicillin and nalidixic resistant colonies) to the total colonies of the recipient strain (ampicillin and nalidixic acid resistant colonies).

For the screening, cultures of donor, recipient and helper in exponential phase (A_{600} of 0.5) were washed to eliminate antibiotics, mixed in a proportion of 1:1:1 in a final volume of 3ml and centrifuged to sediment cells. The pellet was resuspended in 50µl of LB, was patched on LB-agar without antibiotic and incubated over night at 30°C. The patch containing the mating mixture was resuspended in 2 mL of LB, diluted and spread in LB-agar selective medium containing 1% of carboxymethyl cellulose (CMC) to obtain 10^2 to 10^3 cfu of transconjugant cells per plate (15 cm diameter). The plates also contained chloramphenicol (12.5 µg mL^{-1}), nalidix acid (15 µg mL^{-1}), ampicillin (100 µg mL^{-1}), salicylate (1mM) to induce heterologous transcription and arabinose (1mM) to increase the fosmid copy number.

Once colonies were visible, lysis was induced by pouring 8 mL^{-1} of soft agar containing AHT to a final concentration of 0,2µg mL^{-1} and plates were incubated o/n at room temperature. Subsequently, the cellulase activity was revealed by Congo red staining [4]. Each plate was spread with 5 ml of Congo red 1% and incubated at room temperature for 30 minutes. Then, Congo red was removed, 8 ml of 1 M NaCl was added and plates were incubated at room temperature for 30 minutes. Finally, the NaCl was removed, and the stained plates were observed for clear haloes around the colonies.

After detection of positive clones, survivors from these colonies were picked out through the soft agar layer and streaked on LB plates supplemented with CMC 1%, salicylate 1mM, arabinose 1mM and antibiotics and in EBU plates supplemented with antibiotics. One the streak were visible, lysis was induced by pouring AHT in a layer of soft agar in both CMC and EBU plates and plates were incubated for 4-6 hour at 37ºC until lysis was visible in EBU plates. Then, Congo red staining was used for the cellulase activity confirmation on plates containing CMC , as explained above.

For future analysis of the positive clones, they should be transferred by conjugation or electroporation to recipient strains lacking the lysis plasmid.

Supplementary Figure S1:

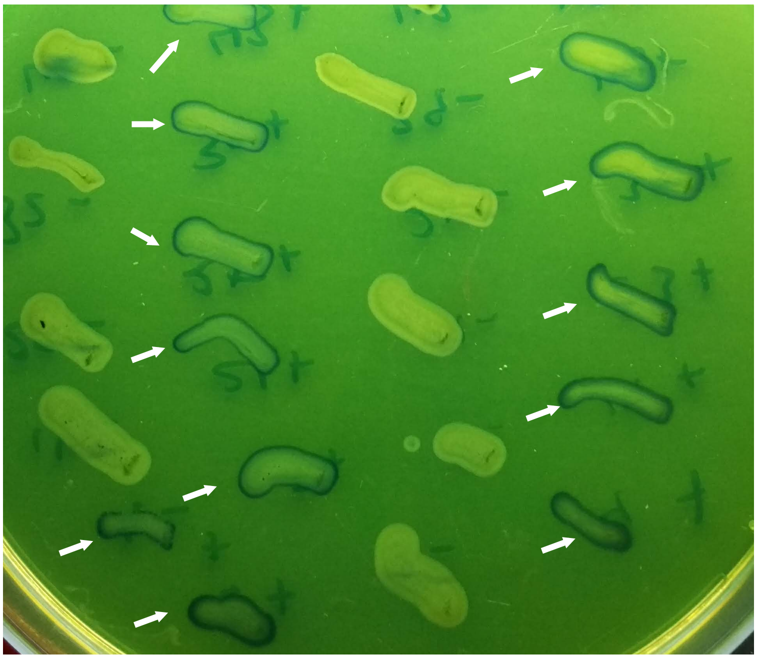

Figure S1: Discrimination between colonies that carry lysis and control vectors. To distinguish between lysis-carrying colonies bearing the lysis and the control vectors after amylase assay or the screening of cellulases, colonies suspected of carrying lysis or control plasmids were picked through the soft agar layer and cultured in EBU plates. One the streak were visible, lysis was induced by pouring AHT in a layer of soft agar over the EBU plates, incubated at room temperature o/n and monitored for lysis. All the colonies suspected of carrying the lysis plasmid turned dark blue (black arrows) while the colonies suspected of having the control plasmids remained white.

Supplementary Table S1. Strain and plasmids

	Characteristics	Reference
***E. coli* strains**		
DH5α	*F⁻ φ80lacZΔM15 Δ(lacZYA-argF)U169 recA1 endA1 hsdR17(rK⁻, mK⁺) gal– phoA supE44 λ⁻ thi-1 gyrA96 relA1*	[5]
EPI300-T1R	*F⁻ mcrA D(mrr-hsdRMS-mcrBC) (StrR) φ80dlacZDM15 DlacX74 recA1 endA1 araD139 D(ara, leu)7697 galU galK l2 rpsL nupG trfA tonA dhfr*	Epicentre
MPO554 Nal^R	*EPI300-T1R Dtrg:: nahR/Psal–gene N* Nal^R	[3]
MPO554 Gm^R	*EPI300-T1R Dtrg:: nahR/Psal–gene N* Gm^R	This work
**Plasmids**		
pASKIBA43plus	Ap^R, expression plasmid. The expression cassette is under transcriptional control of the tetracycline promoter/operator	IBA Biot technology
pMPO1070	Derived from pASKIBA43plus. Ap^R	This work
pMPO1077	pMPO1070 derived plasmid with P_{tet}-*SRRz*	This work
pMPO1631	Cm^R, pWSK29 derived plasmid with P_{bla}-*tetR*	[6]
pMPO1632	pMPO1086 derived plasmid with CmR, P_{tet}-*SRRz* and P_{bla}-*tetR*	[6]
pRK2013	Km^R, helper in triparental matings	[2]
pBBR1MCS5	Gm^R	[7]
pCP20	Ap^R Cm^R Ts (30ºC)	[8]
pMPO579	Cm^R, fosmid expression vector	[3]

REFERENCES

1 Terron-Gonzalez, L., Martin-Cabello, G., Ferrer, M. & Santero, E. Functional Metagenomics of a Biostimulated Petroleum-Contaminated Soil Reveals an Extraordinary Diversity of Extradiol Dioxygenases. *Appl Environ Microbiol* **82**, 2467-2478, doi:10.1128/AEM.03811-15 (2016).

2 Figurski, D. H. & Helinski, D. R. Replication of an origin-containing derivative of plasmid RK2 dependent on a plasmid function provided in trans. *Proc Natl Acad Sci U S A* **76**, 1648-1652 (1979).

3 Terron-Gonzalez, L., Medina, C., Limon-Mortes, M. C. & Santero, E. Heterologous viral expression systems in fosmid vectors increase the functional analysis potential of metagenomic libraries. *Sci Rep* **3**, 1107, doi:10.1038/srep01107 (2013).

4 Carder, J. H. Detection and quantitation of cellulase by Congo red staining of substrates in a cup-plate diffusion assay. *Anal Biochem* **153**, 75-79 (1986).

5 Hanahan, D. Studies on transformation of Escherichia coli with plasmids. *J Mol Biol* **166**, 557-580 (1983).

6 Camacho, E. M., Mesa-Pereira, B., Medina, C., Flores, A. & Santero, E. Engineering Salmonella as intracellular factory for effective killing of tumour cells. *Sci Rep* **6**, 30591, doi:10.1038/srep30591 (2016).

7 Kovach, M. E. *et al.* Four new derivatives of the broad-host-range cloning vector pBBR1MCS, carrying different antibiotic-resistance cassettes. *Gene* **166**, 175-176 (1995).

8 Cherepanov, P. P. & Wackernagel, W. Gene disruption in Escherichia coli: TcR and KmR cassettes with the option of Flp-catalyzed excision of the antibiotic-resistance determinant. *Gene* **158**, 9-14, doi:037811199500193A [pii] (1995).
